# Bilateral patellar tendon rupture following indirect trauma in a young patient due to suspected chronic tendon degeneration caused by repetitive microtrauma: A case report

**DOI:** 10.1016/j.tcr.2026.101385

**Published:** 2026-05-21

**Authors:** Ben Murphy, Nicholas Stratford, Robert Flavin

**Affiliations:** a Department of Trauma & Orthopaedic Surgery, St Vincent's University Hospital, Elm Park, Dublin 4, Ireland

**Keywords:** Bilateral patellar tendon rupture, Extensor mechanism, Tendon degeneration, Ultrasound diagnosis, Case report

## Abstract

**Introduction:**

Bilateral patellar tendon rupture is an uncommon injury, particularly in young, healthy individuals without established risk factors for tendinopathy. Most reported cases involve systemic disease or medication-related tendon compromise. We present a rare case of simultaneous bilateral patellar tendon rupture in a healthy, athletic male following an indirect mechanism of injury.

**Case presentation:**

A 33-year-old man sustained acute bilateral knee injuries while playing recreational rugby. He reported an audible “pop” in both knees, immediate pain, and inability to extend either leg. He had no history of diabetes, connective tissue disease, corticosteroid or fluoroquinolone use, nor other recognized risk factors. Clinical assessment demonstrated inability to straight leg raise bilaterally, with a palpable tendon defect only on the right side. Point-of-care ultrasound suggested bilateral tendon rupture, though diagnostic uncertainty persisted for the left knee due to swelling. The patient underwent urgent surgical repair of the right tendon using a Krackow suture technique with transosseous fixation, followed by delayed repair of the left tendon after departmental ultrasound confirmed rupture. Intra-operatively, both tendons appeared macroscopically degenerative. Postoperative rehabilitation involved bracing, progressive range of motion, and physiotherapy. At 3-month follow-up, he had regained full functional range of motion and returned to unrestricted daily activity.

**Discussion:**

Bilateral patellar tendon rupture in the absence of systemic risk factors is rare and may be underrecognized, especially when clinical signs are obscured. This case highlights the diagnostic value of point-of-care ultrasound in ambiguous presentations and reinforces the importance of early surgical repair and structured rehabilitation for optimal functional recovery.

## Highlights

• Bilateral patellar tendon rupture is rare without predisposing risk factors
• Clinical signs may be obscured by swelling, delaying accurate diagnosis
• Point-of-care ultrasound aids diagnosis when findings are uncertain
• Early repair and structured rehabilitation restore excellent knee function

## Introduction

Bilateral patellar tendon ruptures are rare, especially in patients with no predisposing risk factors for tendinopathy. The majority of published literature on this condition describes patients with pre-existing risk factors for tendinopathy. Here, we present the case of a young, healthy male who suffered bilateral patellar tendon ruptures after an indirect trauma.

## Case report

Our patient was a 33 year old, Caucasian male who sustained bilateral, simultaneous patellar tendon ruptures while engaging in a game of recreational tag rugby. He described feeling a “pop” in both knees with immediate, intense pain that caused him to fall to the ground. From his recollection of the event, his foot positioning at the time of the injury seemed to indicate that both feet were planted in the ground. This resulted in eccentric contraction of his flexed knees.

The patient had no previous medical history such as diabetes mellitus, rheumatoid arthritis or any known connective tissue disorder that might predispose him to tendon injury. He was not taking any medications at the time of the injury. He had no recent or remote history of fluoroquinolone antibiotic use or illicit steroid use. He was a non-smoker. He managed a gym and regularly engaged in high-intensity exercise both as an instructor and as an athlete. Of note, he did mention having recurrent and persistent episodes of knee pain as an adolescent that caused him to cease all competitive, full-contact rugby activities.

**Fig. 1:**
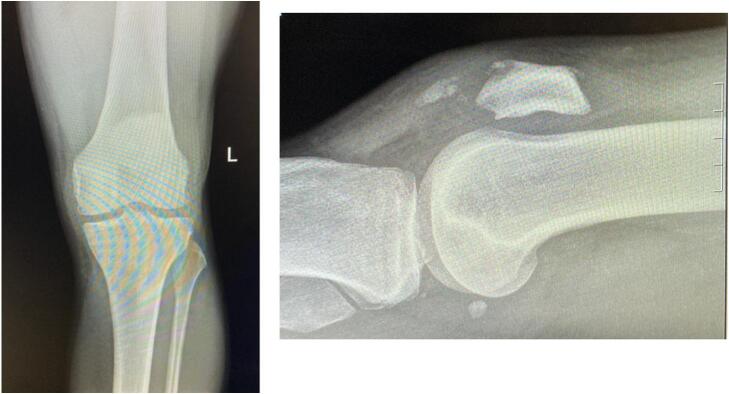
AP and lateral radiograph of left knee.

**Fig. 2:**
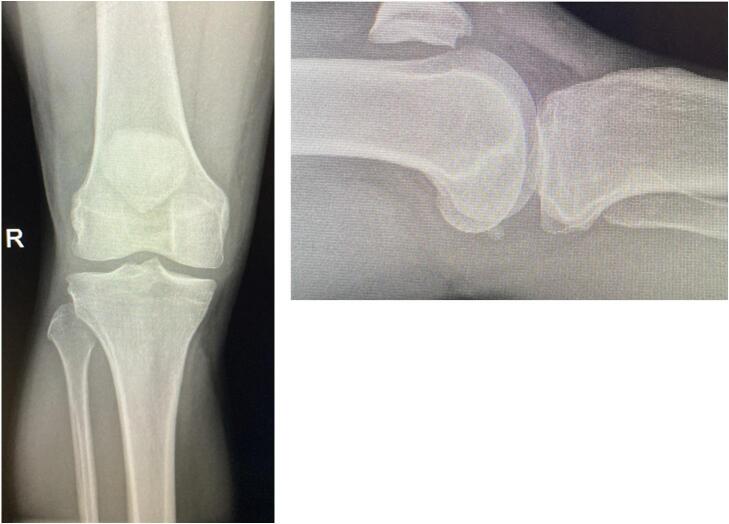
AP and lateral radiograph of right knee.

On arrival in the emergency department, the patient was unable to straight leg raise bilaterally. AP and lateral radiographs of both knees were performed **(**Fig. 1**,** Fig. 2**)**. A point of care ultrasound was performed by the emergency medicine consultant who felt that the patient had suffered a mid-substance tear of both patellar tendons. We, as the orthopaedic service, were then called to review the patient.

The patient was examined by the orthopaedic consultant who agreed that the right patellar tendon was likely ruptured. He could appreciate a palpable gap in the tendon, along with the patient's inability to straight leg raise. The patient's left knee had a significant effusion which made it more difficult to palpate a gap in the tendon. This, coupled with the relative rarity of a potential bilateral patellar tendon rupture diagnosis, informed the decision to obtain further imaging. A decision was made to operate on the right lower limb and a departmental ultrasound scan of the left patellar tendon was booked. This was delayed until two days later due to limited access to ultrasound in our institution out-of-hours at a weekend.

The patient underwent suture repair of his right patellar tendon rupture one day after his injury. A standard midline incision confirmed the diagnosis of a mid-substance patellar tendon rupture. The tendon was noted to be degenerative in appearance. Repair was performed using two number 5 Ticron sutures via the Krackow technique with drilling of three intra-osseous tunnels in the patella. The retinaculum was reinforced with a continuous, locking number 1 Vicryl suture. Closure was performed in the standard fashion using interrupted number 1 Vicryl and 3–0 Nylon for skin closure. The wound was closed in 30 degrees of flexion. A cylinder cast was then applied and the patient was allowed to weight bear on the operative side.

**Fig. 3:**
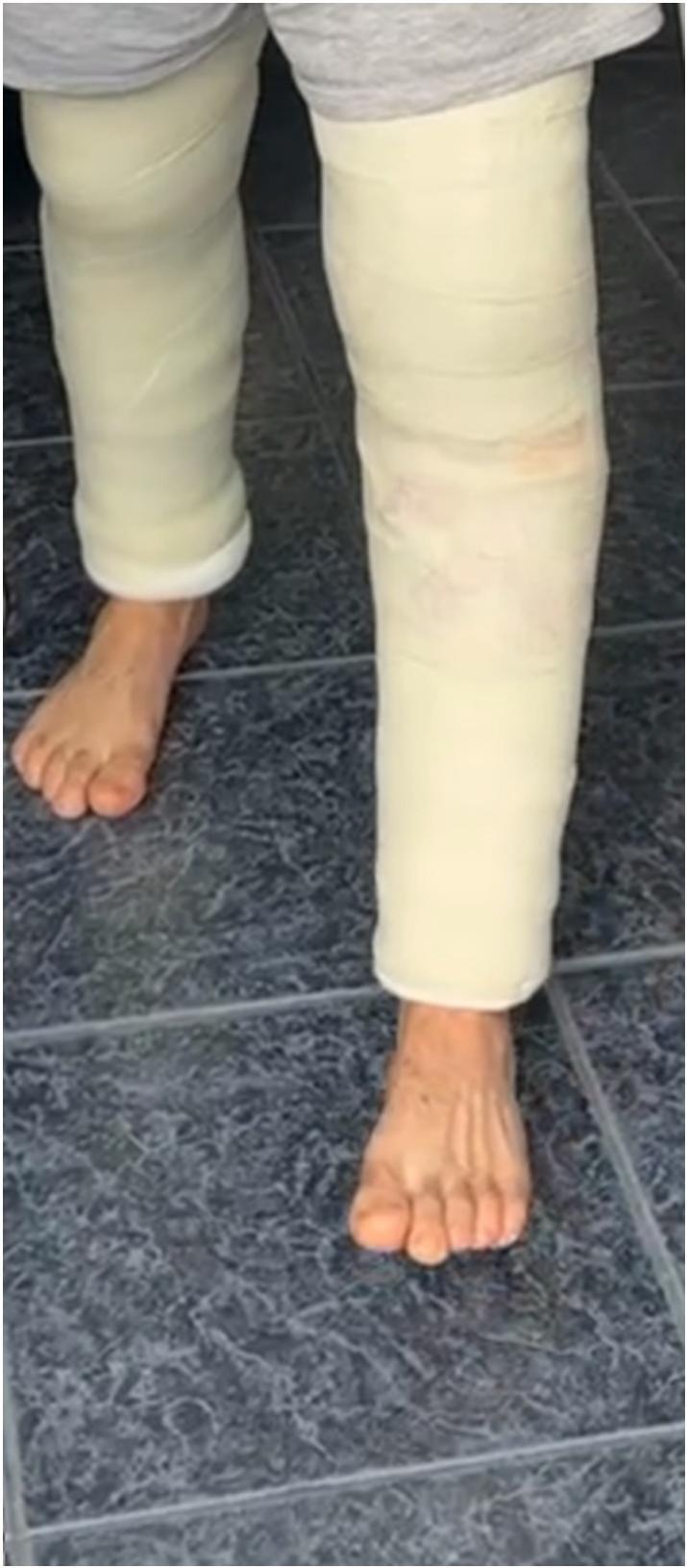
Post-operative clinical image of patient with cylinder casts.

A departmental ultrasound of the patient's left patellar tendon was performed day 1 post-operatively and confirmed that it had also ruptured. The patient's left patellar tendon was then repaired in a similar fashion to that of his right side and a cylinder cast applied **(**Fig. 3**)**. Again, the degenerative appearance of the patient's tendon was noted intra-operatively. He was discharged day 1 post-operatively.

**Fig. 4:**
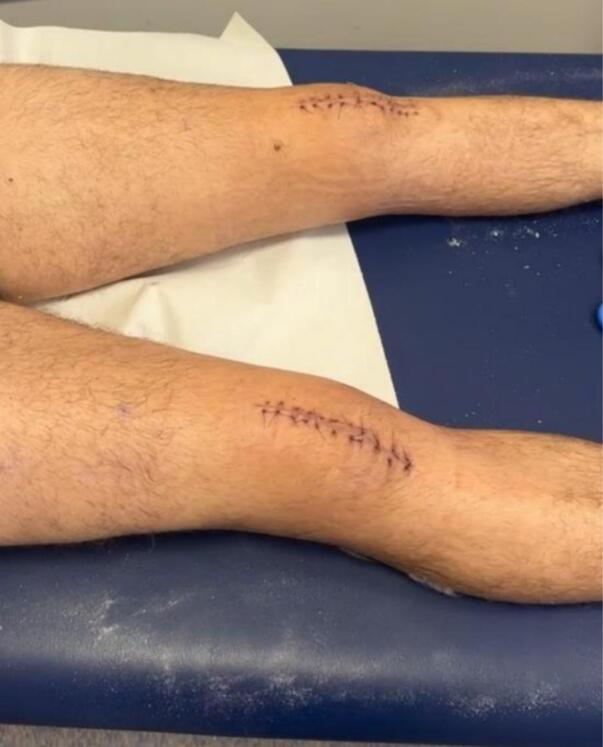
Clinical image from first post-operative appointment showing healed surgical incisions.

**Fig. 5:**
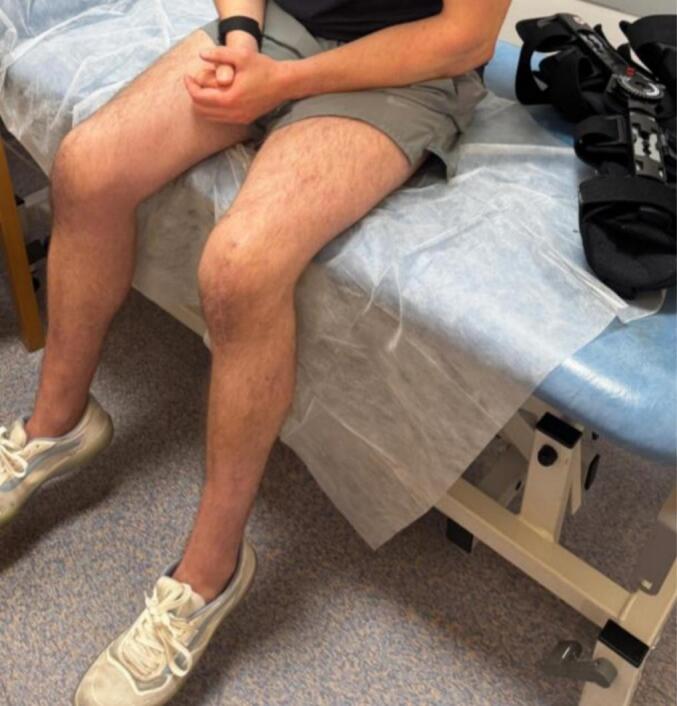
Clinical image of patient with braces removed, healed surgical incisions and knees in nearly 90 degrees of flexion.

His first post-operative assessment came nineteen and sixteen days after his right and left tendon repairs, respectively. His wounds were well-healed and his sutures were removed **(**Fig. 4**).** He was placed into a DonJoy brace bilaterally and was commenced on a gradual range of motion programme with the physio. This programme involved increasing knee flexion in the brace by 30 degrees every two weeks. Unrestricted ROM in the brace was permitted from 8 weeks and at 10 weeks after surgery the braces were slowly weaned **(**Fig. 5**)**. At 3-months follow-up, both knees had a full active ROM 0–120° and our patient was mobilising without limitation. Despite bilateral quadriceps wasting, our patient had resumed all daily activities. Our patient was advised to continue supervised physiotherapy and return to sport gradually after 6–8 months. At 12-month follow-up, the patient had returned to full occupational and recreational sporting activities without limitation. Clinical examination demonstrated full bilateral knee range of motion with no extensor lag. He reported no ongoing pain, instability or functional restriction. Functional outcome assessment using the Lysholm Knee Score demonstrated excellent outcomes, with scores of 96/100 in the right knee and 95/100 in the left knee.

## Discussion

The patellar tendon plays a vital role in the extensor mechanism which then has an essential role in gait. The extensor mechanism consists of the quadriceps muscles and their tendon, the patella and the patellar tendon, which attaches to the tibial tuberosity. Rupture of the patellar or quadriceps tendon or a fracture of the patella itself can disrupt the extensor mechanism [1].

Ruptures of the patellar tendon are more commonly unilateral and are more prevalent in athletic adults 40 years of age or younger, similar to our patient. If one's patellar tendon is healthy and without signs of degeneration, then the patella bone itself would be considered the weakest component of the extensor mechanism. The patella usually fails in tension, leading to a transverse fracture [2]. As in this patient, the usual mechanism of injury is knee flexion combined with eccentric quadriceps contraction. Huberti et al. described the concept of the extensor mechanism force ratio, which draws an association between the degree of knee flexion and the probability of tendon rupture. This ratio is the fraction of force found in the patellar tendon divided by the quadriceps tendon. The ratio is greater than 1.0 with knee flexion less than 45 degrees, putting the quadriceps tendon at a higher risk of injury [3]. With the knee in greater than 45 degrees of flexion, the patellar tendon is at a higher risk of failure.

Although our patient had no typical risk factors for patellar tendon rupture, the gross appearance of his patellar tendons intra-operatively was indicative of chronic tendon degeneration. A normal, healthy patellar tendon requires a huge force to fail and should not rupture under physiological loads, in the absence of well-recognized risk factors [4]. To rupture a normal patellar tendon, it is estimated that 17.5 times a patient's body weight is required [5].

Common risk factors for tendon rupture, none of which were present in this patient, include conditions such as systemic lupus erythematosus (SLE), diabetes mellitus, rheumatoid arthritis, chronic renal failure and corticosteroid use [6]. We know these conditions cause patients to be more susceptible to patellar tendon rupture as they are known to weaken collagenous structures [7], [8]. Spontaneous rupture of a tendon, that is, in the presence of minimal trauma with no predisposing risk factors, has been defined in the literature. The rupture is said to occur during movement and activity that should not and usually does not damage the involved musculotendinous units [9]. Our patient's history of recurrent knee pain limiting sporting activity as an adolescent, coupled with his occupation involving high-intensity and high-impact exercise suggests long-standing chronic tendon degeneration, secondary to repetitive microtrauma – thus resulting in spontaneous tendon rupture.

Bilateral patellar tendon ruptures are a rare entity and so present a diagnostic challenge. Clinical suspicion for these injuries is usually low and thus they have the potential to be missed diagnoses. Admittedly as the orthopaedic service on-call, we had some clinical doubt with regards the referral we received and suspected that the point-of-care ultrasound was not entirely accurate. Our case highlights the role that this investigation may play in future diagnoses of this condition if there is any clinical doubt, as was the case in Ogle et al.'s report [10]. Point-of-care ultrasound is advantageous due to its portability, low cost and lack of ionising radiation. Tendon injuries are especially amenable to ultrasound due to the superficial location of their structures [11], [12]. Indeed, the diagnosis of even unilateral patellar tendon ruptures can be a challenge, with initial misdiagnosis rates of up to 38% quoted in the literature [13]. The presence of severe haematoma or swelling that accompanies acute ruptures may conceal important clinical signs. This was the case in our patient's left-sided injury where the palpable infrapatellar gap was not appreciated and introduced some diagnostic uncertainty. It is of utmost importance that early diagnosis of patellar tendon ruptures is established [6]. Patellar tendon ruptures require operative intervention to prevent disability, with prompt recognition and surgical management improving outcomes by preventing scarring, retraction and decreased function [14]. In proximal avulsions of the tendon, as in our patient, primary repair can be achieved using a transosseous suture technique [15].

## Conclusion

Bilateral patellar tendon ruptures are a rare presentation. They are even less common in those without pre-existing known risk factors for rupture. As a result of this, they can present a diagnostic challenge. Prompt diagnosis leads to improved outcomes for patients and ensures an appropriate return of function. Clinical examination is the cornerstone of diagnosis, aided by lateral radiographs of the knee and may be augmented by point-of-care ultrasound if there is still clinical uncertainty. Early surgical intervention and guided post-operative rehabilitation is the standard of care. In presenting our patient, we hope to raise awareness of this condition, thereby improving diagnostics and facilitating improved patient outcomes.

## CRediT authorship contribution statement

**Ben Murphy:** Writing – review & editing, Writing – original draft, Methodology, Formal analysis. **Nicholas Stratford:** Writing – review & editing, Methodology, Formal analysis. **Robert Flavin:** Writing – review & editing, Supervision, Methodology, Formal analysis.

## Funding

This research did not receive any specific grant from funding agencies in the public, commercial, or not-for-profit sectors.

## Declaration of competing interest

The authors declare that they have no known competing financial interests or personal relationships that could have appeared to influence the work reported in this paper.
